# Reverse dynamics analysis of contact force and muscle activities during the golf swing after total hip arthroplasty

**DOI:** 10.1038/s41598-023-35484-y

**Published:** 2023-05-29

**Authors:** Tetsunari Harada, Satoshi Hamai, Daisuke Hara, Tsutomu Fujita, Kazuya Okazawa, Naoya Kozono, Shinya Kawahara, Ryosuke Yamaguchi, Masanori Fujii, Satoshi Ikemura, Goro Motomura, Yasuharu Nakashima

**Affiliations:** 1 Department of Orthopaedic Surgery, Faculty of Medical Sciences, Kyushu University, 3-1-1 Maidashi, Higashi-ku, Fukuoka, 812-8582 Japan; 2 Department of Medical-Engineering Collaboration for Healthy Longevity, Kyushu University, 3-1-1 Maidashi, Higashi-ku, Fukuoka, 812-8582 Japan; 3 Department of Rehabilitation, Faculty of Medical Sciences, Kyushu University, 3-1-1 Maidashi, Higashi-ku, Fukuoka, 812-8582 Japan; 4 Department of Orthopaedic Surgery, Faculty of Medical Sciences, Saga University, 5-1-1 Nabeshima, Saga, 849-0937 Japan

**Keywords:** Pain, Outcomes research, Physics

## Abstract

There are no reports on hip kinetics including contact forces and muscle activities during the golf swing after total hip arthroplasty (THA). The aim of this study was to identify the characteristics of three-dimensional dynamics during the golf swing. Ten unilateral primary THA patients participated in motion capture test of their driver golf swing. The driver swing produced approximately 20–30° of rotation in both lead and trail replaced hips. The mean hip contact forces (HCFs) of lead and trail replaced hips were 5.1 and 6.6 × body weight, respectively. Left and right THAs showed similar HCFs of lead and trail hips. More than 60% of the Percent maximum voluntary isometric contraction was found in bilateral iliopsoas muscles in all unilateral THA. Three factors [female sex, lower modified Harris Hip Score, and higher HCF of surgical side] were associated with the golf-related replacement hip pain. Golf is an admissible sport after THA because driver swings do not contribute excessive rotation or contact forces to hip prostheses. HCF could be reduced through swing adjustments, which may allow patients with golf-related replacement hip pain to develop a comfortable golf game free from pain.

## Introduction

Total hip arthroplasty (THA) is one of the most successful orthopedic procedures^1^. Golf is a globally popular sport with participants from a broad range of ages with a significant portion having a hip replacement (1–9.5%)^2–4^, making it the most common sport followed by walking, swimming, gymnastics, and cycling^5,6^. While golf is not considered to be a high-impact sport and the majority of attending surgeons recommend a return to golf after THA, no previous study has addressed the kinetic data of golfers performing a drive after THA^7^.

D’Lima et al. showed that a golf swing produces an in vivo contact force of 4.5 × body weight (BW) on the lead knee and 3.2 × BW on the trailing knee in total knee arthroplasty (TKA) patients, similar to the peak load during jogging^8^. The percentage of TKA patients that felt mild pain during and after playing golf were 16% and 35%, respectively. The pain was greater in the lead knee because of the higher force applied to the lead knee while hitting the ball^9^. Mallon et al. recorded that 11% and 41% of THA patients have mild pain during and after playing, respectively^10^. A recent study reports that 98% of patients return to golf after THA, with an improvement in pain visual analog scale (VAS) from 6.4 to 2.5 while playing^11^.

Using image-matching techniques, Hara et al. have demonstrated that the golf swing does not produce excessive hip rotation or cup-head translation after THA. However, there are no reports on hip kinetics including contact force in the golf swing after THA^12^.

The present study aimed to address: (1) three-dimensional (3D) motion including hip range of motion (RoM), (2) HCF and hip muscle activation, and (3) the influencing factors for replaced hip pain related golf actions. Query ID="Q4" Text="Please note we have moved the section “Ethics approval and consent to participate, Informed consent” to the end of the methods, as per house style".

## Methods

### Patients

All patients (n = 1197) underwent primary cementless THA in our institution between January 2008 and December 2018. Among them, 766 satisfied each of the following inclusion criteria: (1) alive at the time of the survey and (2) evaluation by a surgeon within the past two years. A review of medical records revealed that 17 patients (2.2%) were recreational golfers. Of the 17 golfers, the following were excluded: three patients with bilateral THAs, one patient hospitalized with a medical condition, one with back pain that made swinging difficult, and one declined participation; the remaining 10 patients signed an institutional review board-approved (IRB Number: 2019–323) informed consent. All THAs were performed using a posterolateral approach, with a uniform protocol for postoperative rehabilitation^13^.

### Radiographic data

Leg length difference (LLD) and global femoral offset (GFO) before and after THA were assessed using anteroposterior radiographs of the pelvis as described previously^14,15^.

### Patient reported outcome measurements

VAS pain scores in which a respondent selects a whole number (0–10) while playing and after playing golf, and the Modified Harris Hip Score (mHHS) were assessed during the dynamic capturing of the golf swing^16,17^.

### Data capture

**Figure 1:**
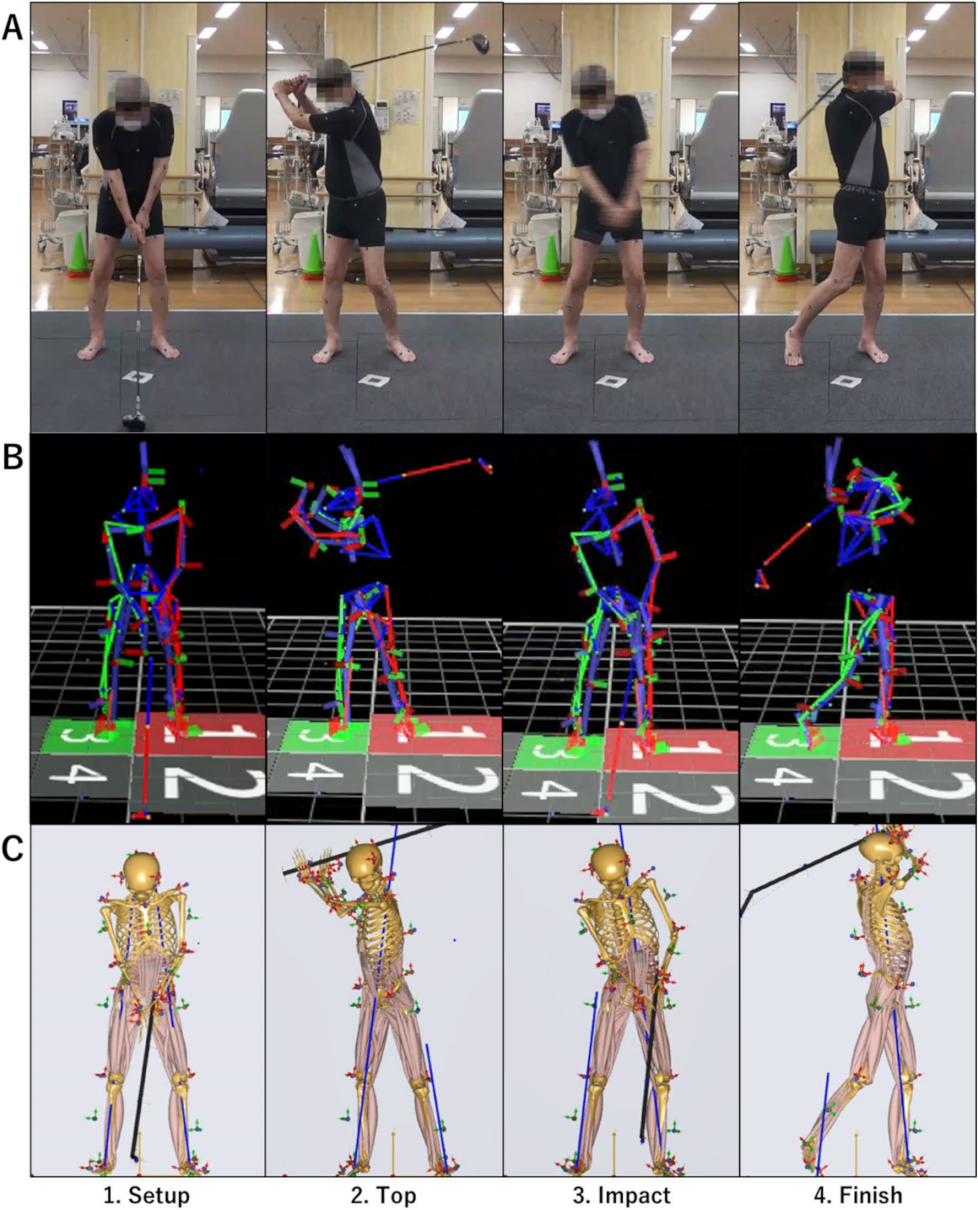
Patient's (recreational right-handed golfer) driver golf swing. (**A**) Actual driver swing scene, (**B**) Motion capture system (VICON), (**C**) Musculoskeletal modeling system (AnyBody).

A 10-camera VICON motion capture system with a sampling frequency of 200 Hz (VICON, Oxford Metrics Group, UK) and two force plates with a sampling frequency of 1000 Hz (AMTI, Waterton, MA, USA) were used (Fig. 1). Each patient was provided with form-fitting shorts and a shirt during testing; patients were barefoot during testing to control for footwear-associated changes in the ground reaction forces (GRF)^18^. Reflective markers were placed on the full body in accordance with the Plug-in Gait configuration^19^, with additional markers on the lateral foot and anterior thigh and shank; in addition, a total of six markers were placed—three on the club head and three on the shaft of the driver club, respectively; the club head markers were used to identify the phases of the golf swing^20^. Each patient was asked to perform a drive swing without a ball using the 285 g driver club (Head, HONMA Twin Marks MG410; Shaft, HONMA ARMRQ 851). After a minimum of three practice trials, patients repeated the motions until three trials suitable for data analysis were obtained.

### Data processing

The golf swing is divided into four events (setup, top, impact, and finish; Fig. 1). Maximum club head speed was calculated from the club head markers around impact. The marker trajectories and the GRF data were low pass filtered using a Butterworth filter with a cutoff frequency of 20 Hz and 12 Hz, respectively. The hip joint angle was calculated using the Plug-in Gait model with Cardin rotational sequence of flexion–extension, adduction-abduction, and internal–external rotation (Fig. 1).

The musculoskeletal model used in this study is a standard model (v.2.3.4, AMMR, MoCapModel) available in the AnyBody Modeling System (v.7.3.4, AnyBody Technology, Denmark). The hip joint was modeled as a spherical joint with three rotational degrees of freedom. The knee, ankle, and subtalar joints were simulated as hinge joints. A detailed musculoskeletal model based on the cadaveric data set by Carbone et al^21^. was scaled to each patient's body size, based on marker data collected in the setup position. For fat scaling, the body mass index (BMI) was taken into account according to the equation proposed by Frankenfield et al^22^.

Marker trajectories and the GRF data for each trial were used as inputs for inverse kinematic analysis based on third-order polynomial muscle mobilization calculate HCF and muscle force^23–26^ (Fig. 1). HCF was evaluated using a value criteria to normalized by BW ratio. Percent maximum voluntary isometric contraction (%MVIC) is defined as muscle force divided by maximal muscle strength for a particular muscle at a particular instant in time^27^. The muscles around the hip joint [iliopsoas, gluteus maximus, gluteus medius, gluteus minimus, rectus femoris, biceps femoris long head, medial hamstring (semimembranosus and semitendinosus), adductor magnus, and adductor longus] with greater than 40% of %MVIC during the golf swing were extracted^28,29^.

### Statistical analysis

Continuous variables were expressed as mean ± standard deviation (range or 95%CI). Statistical analysis was performed using JMP software v.14.0 (SAS Institute, Cary, NC). The Wilcoxon rank-sum test was used to compare demographic data [age, height (Ht), BW, BMI, follow-up duration, LLD, GFO, mHHS, average score, handicap, and VAS pain score] and kinematics and kinetics data [maximum club head speed, hip range of motion (RoM), and HCFs and %MVIC of muscles around hip joint] between left THA and right THA patients, along with the same factors between painful and painless patients during and after playing golf. The chi-square test was used to compare sex and diagnosis. Statistical significance was set as *P* < 0.05. Power analyses in the HCF difference between left THA and right THA and between with and without hip pain showed that the combined sample size of the two groups were 8 (4 and 4 patients) and 10 (2 and 8 patients) which provided 80% statistical power to detect the 1.6 difference in HCF between the two groups for a ratio one-to-one and one-to-four, respectively. This assumes that the probability value is < 0.05 and the standard deviation is 0.7^30^.

### Ethics approval and consent to participate

This study was approved by the Clinical Research Ethics Review Committee, Department of Medical Sciences, Kyushu University (IRB Number: 2019-323). All patients gave informed consent for the use of their demographic data, clinical score data, dynamic data, and images in motion in the study. The study was carried out in accordance with the principles of the Helsinki declaration. All authors agree to participate and consent to the manuscript.

### Informed consent

Informed consent obtained from all study participants for publishing identifying information/image for this study.

## Results

### Patient demographics

Demographic characteristics (sex, preoperative diagnosis, age, height, weight, postoperative follow-up duration, GFO, LLD, mHHS, average score, and handicap) were similar between L-THA and R-THA patients (*P* > 0.05; Table 1). There were two patients (1 left THA and 1 right THA) with mild pain in the surgical hip during and after playing golf; VAS pain score in and after golf were also similar between L-THA and R-THA patients (*P* > 0.05; Table 1).

**Table 1:** Demographic data.

	Patient number										Patient group			*P* value
	1	2	3	4	5	6	7	8	9	10	All THA (N = 10)	Left THA (N = 5)	Right THA (N = 5)	
Surgical side	Left	Left	Left	Left	Left	Right	Right	Right	Right	Right				
Sex	Female	Male	Female	Male	Male	Male	Male	Male	Male	Female	7 male/3 female	3 male/2 female	4 male/1 female	0.49
Diagnosis	OA	OA	OA	ONFN	OA	OA	ONFN	OA	OA	OA	8 OA/ 2 ONFN	4 OA/ 1 ONFN	4 OA/ 1 ONFN	1
Age (y)	58	78	56	68	66	75	72	69	66	72	68 ± 7	65 ± 9	71 ± 4	0.25
Height (cm)	148	172	157	179	174	170	168	161	162	157	165 ± 9	166 ± 13	164 ± 5	0.46
Body weight (kg)	49	72	52	76	67	65	79	69	78	67	68 ± 10	63 ± 12	72 ± 7	0.25
Follow-up (months)	84	97	79	36	146	146	36	29	122	30	81 ± 47	89 ± 40	73 ± 57	0.46
LLD (mm)	12	2	3	2	6	3	5	10	8	2	5 ± 4	5 ± 4	6 ± 3	0.46
Difference in GFO (mm)^a^	1	-1	-4	-4	21	25	2	25	8	-9	6 ± 13	3 ± 10	10 ± 15	0.25
mHHS	95.6	95.6	91.2	97.8	97.8	95.6	95.6	100	97.8	93.4	96 ± 3	96 ± 3	96 ± 3	0.74
Average score	130	110	90	95	100	100	80	100	125	100	103 ± 15	105 ± 16	101 ± 16	0.83
Handicap	45	26	14	17	20	21	8	21	36	20	23 ± 11	24 ± 12	21 ± 10	0.83
VAS pain score in golf	0	0	2	0	0	0	0	0	0	3	0.5 ± 1.1	0.4 ± 0.9	0.6 ± 1.3	0.88
VAS pain score after golf	0	0	4	0	0	0	0	0	0	3	0.7 ± 1.5	0.8 ± 1.8	0.6 ± 1.3	0.88

THA, total hip arthroplasty; LLD, leg length discrepancy; GFO, global femoral offset; mHHS, modified Harris Hip Score; VAS, visual analog scale; OA, osteoarthritis; ONFH, osteonecrosis of the femoral head.

Continuous variables are expressed as mean ± standard deviation.

*P* value for the comparison of demographic data between left and right THAs (*P* < .05).

^a^Positive difference in GFO indicates that the postoperative GFO is more lateralized than the contralateral GFO.

### Kinematics and kinetics data

**Figure 2:**
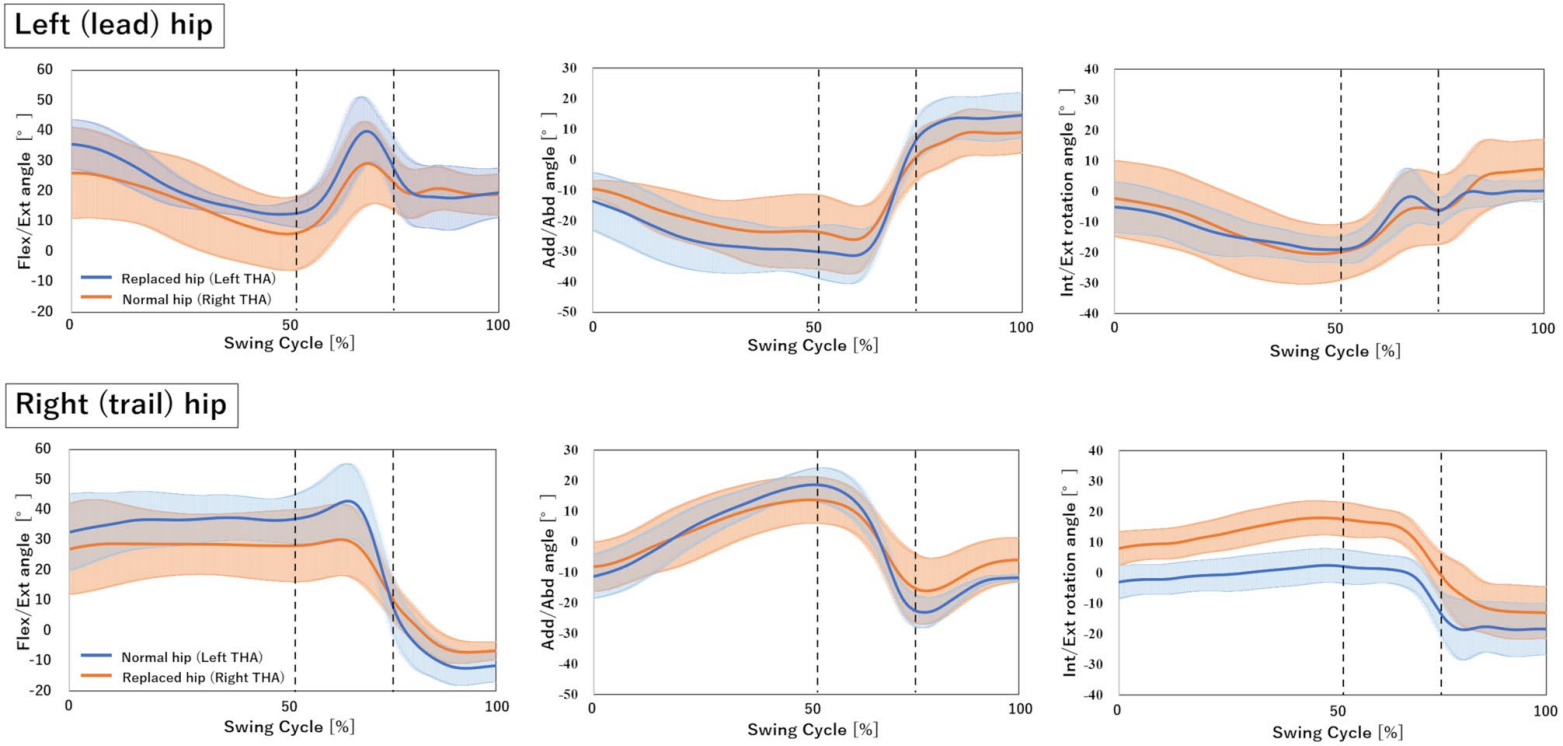
Hip range of motion. Blue solid line, Left THA; Orange solid line, Right THA; First black dotted line, Top; Second black dotted line, Impact.

The mean maximum club head speed and the mean left and right hip RoMs of 3D were no significant difference between left THA and right THA (Table 2 and Fig. 2).

**Table 2:** Kinematics and kinetics data.

	Patient number										Patient group			*P* value
	1	2	3	4	5	6	7	8	9	10	All THA (N = 10)	Left THA (N = 5)	Right THA (N = 5)	
Surgical side	Left	Left	Left	Left	Left	Right	Right	Right	Right	Right				
Club Head Speed (m/s)	24	25	28	43	37	32	39	34	28	20	31 ± 7	31 ± 8	31 ± 7	0.92
Flex-Ext RoM (°, L)	24	29	44	36	38	19	24	33	45	11	30 ± 11	34 ± 8	26 ± 13	0.35
Flex-Ext RoM (°, R)	42	49	73	59	62	45	55	32	49	22	49 ± 15	57 ± 12	41 ± 13	0.08
Add-Abd RoM (°, L)	51	35	62	46	45	41	61	36	36	12	42 ± 15	48 ± 10	37 ± 18	0.25
Add-Abd RoM (°, R)	31	34	54	46	47	27	51	24	37	13	36 ± 13	42 ± 10	30 ± 14	0.17
Int-Ext rotation RoM (°, L)	18	15	31	27	24	28	20	35	39	21	26 ± 8	23 ± 7	28 ± 9	0.25
Int-Ext rotation RoM (°, R)	16	16	26	32	28	33	38	25	40	25	28 ± 8	23 ± 7	32 ± 7	0.17
HCF (N/BW, L)	5.3	2.2	8.2	7.4	4.6	3.9	7.1	5	4.6	2.9	5.1 ± 1.9	5.5 ± 2.4	4.7 ± 1.6	0.6
HCF (N/BW, R)	6.9	3.8	7	7.6	7.3	4.8	7.1	7.9	5.8	8	6.6 ± 1.4	6.5 ± 1.6	6.7 ± 1.4	0.35
Iliopsoas (%, L)^a^	49	38	352	210	165	52	221	143	48	15	129 ± 109	163 ± 129	96 ± 85	0.46
Iliopsoas (%, R)^a^	76	26	165	100	121	35	175	121	61	24	90 ± 55	98 ± 52	83 ± 64	0.6
Biceps femoris long head (%, R)^a^	34	23	35	47	40	19	40	72	58	41	41 ± 16	36 ± 9	46 ± 20	0.35
Medial hamstring (%, R)^a^	31	16	28	30	30	18	29	58	40	107	38 ± 27	27 ± 6	50 ± 35	0.25

THA, total hip arthroplasty; RoM, range of motion; HCF, hip contact force; BW, body weight; %MVIC, percentage maximum voluntary isometric contraction.

Continuous variables are expressed as mean ± standard deviation.

*P* value for the comparison of kinematics and kinetics data between left and right THAs (*P* < .05).

^a^%MVIC were listed when the hip muscles had %MVIC > 40% during golf swing^26,27^.

**Figure 3:**
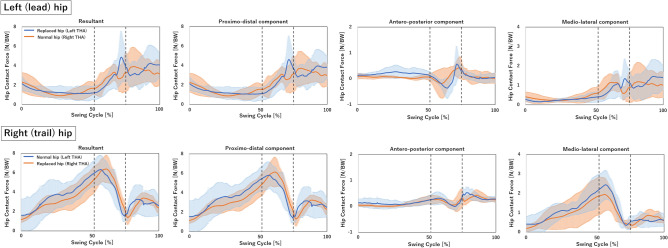
Hip contact force (HCF). Blue solid line, Left THA; Orange solid line, Right THA; First black dotted line, Top; Second black dotted line, Impact.

**Figure 4:**
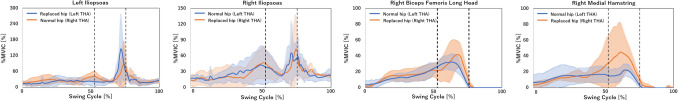
Percentage maximum voluntary isometric contraction (%MVIC). Blue line, Left THA; Orange line, Right THA; First black dotted line, Top; Second black dotted line, Impact.

There was no significant difference in HCF of lead and trail hips between left THA and right THA, with a tendency for a larger HCF of trail hips in unilateral THA (6.6 N/BW vs 5.1 N/BW, *P* = 0.06; Table 2 and Fig. 3). The %MVIC of the iliopsoas muscles in the left THA and right THA patients were more than 80% both on the surgical and contralateral sides (Table 2 and Fig. 4). The biceps femoris long head and medial hamstring of the surgical side in left and right THA patients were less and more than 40%, respectively (Table 2 and Fig. 4). The %MVIC of these hip muscles showed no significant difference between left THA and right THA (Table 2).

### Difference of demographics, kinetics, and kinetics data between patients with and without golf-related replaced hip pain

Patients without replaced hip pain had a significantly higher proportion of males, higher mHHS, and lower HCF of the surgical side compared to patients with pain [seven male and one female vs. zero male and two female, 97.0 ± 1.6 vs. 92.3 ± 1.6 and 5.6 ± 1.8 × BW vs. 8.1 ± 0.1 × BW; *P* = 0.02, 0.03, and 0.04, respectively]. The other demographics, kinetics, and kinetics data showed no significant difference (*P* > 0.05; Table 3).

**Table 3:** Comparison of demographic, kinematics and kinetics Data between the patients with and without replaced hip pain.

	Painful patients (N = 2)	Painless patients (N = 8)	*P* value
Male/female (n)	0/2	07-Jan	0.02
Diagnosis (OA /ONFN, n)	2/0	06-Feb	0.43
Replaced hip side (L/R)	01-Jan	04-Apr	1
Age (y)	64 ± 11	69 ± 6	0.51
Height (cm)	157 ± 0	167 ± 10	0.12
Body weight (kg)	60 ± 11	70 ± 10	0.3
Follow-up (months)	55 ± 35	87 ± 49	0.3
LLD (mm)	4 ± 1	6 ± 4	0.3
Difference in GFO (mm)^a^	− 6 ± 4	10 ± 12	0.07
mHHS	92.3 ± 1.6	97.0 ± 1.6	0.03
Average score	95 ± 7	105 ± 16	0.35
Club Head Speed (m/s)	24 ± 6	33 ± 7	0.19
Flex-Ext RoM (°)	33 ± 38	38 ± 11	0.43
Add-Abd RoM (°)	38 ± 35	39 ± 10	1
Int-Ext rotation RoM (°)	28 ± 4	27 ± 9	1
HCF (N/BW)	8.1 ± 0.1	5.6 ± 1.8	0.04
Iliopsoas (%)	188 ± 232	107 ± 70	1
Biceps femoris long head (%)	35 ± 7	32 ± 23	0.43
Medial hamstring (%)	63 ± 61	23 ± 18	0.19

OA, osteoarthritis; ONFH, osteonecrosis of the femoral head; LLD, leg length discrepancy; GFO, global femoral offset; mHHS, modified Harris Hip Score; RoM, range of motion; HCF, hip contact force; BW, body weight; %MVIC, percentage maximum voluntary isometric contraction. Continuous variables are expressed as mean ± standard deviation.

^a^Positive difference in GFO indicates that the postoperative GFO is more lateralized than the contralateral GFO.

*P* value for the comparison of demographic, kinematics and kinetics data between left and right THAs (*P* < .05).

## Discussion

This in vivo study for the first time examined kinematics and kinetics of the hip joint during driver golf swings of patients who had undergone unilateral THA. This study evaluated different parameters including HCF and muscle activation of muscles that exhibited more than 40% of the %MVIC in recreational golfers after unilateral THA, in order to examine the differences in hip loading and muscle activation between left and right THA and between with and without golf-related pain. During the driver swing at a mean maximum head speed of 31 m/s, the mean replaced hip rotations were approximately 20–30° and mean HCFs were 5 and 6.6 × BW at the lead and trail replaced hips, respectively. More than 60% of the %MVIC was found in both iliopsoas muscles, regardless of the surgical side. Being female, lower mHHS, and higher HCF of surgical side were associated with golf-induced pain of the replaced hip.

The 31 m/s of mean maximum head speed in unilateral THA patients was equivalent to that of a similar aged healthy cohort: mean 28.3–38.9 m/s^31–33^. An approximate mean of 20–30° of hip rotation during the driver swing in patients who underwent THA is also equivalent to that of the golf swing analyzed by the motion capture methods in normal hips: mean 20–60°^34,35^. As in a previous study^12^, the driver swing had comparable lead and trail hip rotations regardless of the surgical side, and no excessive deviation of rotational balance. Hip rotation during the golf swing of similar aged THA patients analyzed by the image-matching method was approximately 50°^12^, which is greater than the 20–30° in the present study. The results may have been influenced by differences in the club used (grip only vs. driver club) and analysis method (image matching vs. motion capture)^36,37^. The motion capture system was reported to underestimate hip rotation by approximately 20° in motion involving large hip rotation compared to shape matching technique^37^. However, hip rotation during the golf swing was not an excessive RoM of the Int-Ext rotation, after accounting for this underestimation. RoMs of the Flex-Ext and Add-Abd during the driver swing in this study were similar to those of the golf swing in healthy participants^34,35^. In other words, club head speed and 3D hip RoMs were comparable to those of healthy participants, suggesting that THA had a positive effect on golf performance, including distance and handicap; this is supported by previous reports that most patients are able to play golf after THA, and mostly with an improvement in performance^1,10,11,38^.

Advances in computational techniques using musculoskeletal modeling systems have shown the comparability of estimating hip contact force (HCF) data to in vivo HCF^23,24^. The use of a musculoskeletal modeling system has the advantage that data can be safely collected from a larger number of cases rather than using implants with built-in devices to measure loading data in THA. The mean HCFs of the lead and trail replaced hips during the golf swing (5.5 and 6.7 × BW, respectively) were comparable, with a tendency of larger HCF of trail hips in both left THA and right THA, which was somewhat lower than the HCF while jogging at 6–8 km/h: 5.4–8.5 × BW^30,39^. Although jogging has been classified as a high-impact sport, it has been reported to have no adverse effect on mid-term survival outcome in THA^5,40^. The number of golf swing motions is much less than the number of steps taken while jogging and the repetition of loading cycles is significantly smaller. In addition, no negative effects on mid-term survival rate of implants have been previously observed in golfers^11,41^. Thus, it is suggested that the golf swing is an acceptable motion in patients with unilateral THA, although it produces a high HCF for both lead and trail replaced hips, comparable to that of jogging. There have been no previous reports of HCF in normal participants and THA patients during a golf swing, the proximo-distal component of HCF showed a similar pattern and magnitude as the resultant HCF, consistent with the reported HCF of daily activities in THA patients^23,24^.

The musculoskeletal modeling system can estimate the activation of each muscle around the hip joint which is in agreement with the measured activation^25,26^. It has been reported that more than 60% of MVIC intensity should be used for effective muscle strengthening and that the range should be at least 40–60% to stimulate muscle strength adaptation^28,29^. The driver swing may be useful as an iliopsoas strengthening exercise, because the %MVIC of the bilateral iliopsoas muscles is greater than 60%. Marta et al. demonstrated that the driver swing activated hip extensors in the order of strength of the biceps femoris long head, gluteus maximus, and semimembranosus in the lead leg (51–83%) and gluteus maximus, biceps femoris long head, and semimembranosus in the trail leg (67–100%), indicating a higher muscle activity of the trail leg^42^, which was consistent with the present study, where more than 40% of %MVIC was found in the hip extensors (the biceps femoris long head and medial hamstring) of the trail leg, except in the bilateral iliopsoas muscles. However, overall, %MVIC values were higher in the Marta et al. report^5^ than in the present study, which may have been influenced by differences in the participants (mean 36-year-old healthy volunteers vs. mean 68-year-old THA patients), handicap (< 5 vs. 8–45), and analysis methods (electromyogram vs. musculoskeletal modeling method). The biceps femoris long head and medial hamstring on the right side had approximately 40% of MVIC. Therefore, opposite-direction driver swings may be required to provide a level of muscle activation that balances the bilateral biceps femoris long head and medial hamstring muscular adaptations.

A significantly higher proportion of males were found in the patients without golf-related replaced hip pain compared to patients with pain, consistent with previous reports on the association between sex and persistent pain after THA^43,44^. A significantly higher proportion of males were found in the patients without golf-related replaced hip pain compared to patients with pain, consistent with previous reports on the association between sex and persistent pain after THA^9^. Of golf swings, the minimalist golf swing and partial golf swing are designed to reduce the loading that hips experience throughout the swing^43,45,46^. The minimalist golf swing, which requires torso rotation to be completed in a more upright position during the set-up, results in less hip extension and abduction moments while maintaining golf performance^47^. The partial golf swing, which is adjusted to approximately 80% of full swing distance, requires limiting the natural weight shift of the swing to reduce lateral motion, resulting in a significant reduction in the peak horizontal ground reaction force of both legs^46,47^. This reduction in joint loading resulting from swing adjustments may provide relief from hip pain during and after playing golf.

There are several limitations to the present study. First was the limitation of the small number of study patients. Although power analysis is done, number could be insufficient for groups with and without hip pain. A larger number of patients might have revealed further kinematic and kinetic differences. In addition, we recruited recreational golfers from unilateral primary THA patients; therefore, findings in this study cannot be generalized to amateur or professional golfers. Furthermore, we only analyzed swings using a driver for our data collection. Therefore, we cannot make inferences as to kinematics and kinetics produced using other clubs. Finally, the patients performed their golf swing while barefoot and without a golf ball, which could have altered swing mechanics.

## Conclusion

The driver swing of unilateral THA patients did not contribute excessive hip contact forces to the right and left hip prosthesis, with a maximum club head speed and hip RoMs comparable to those of healthy participants. The driver swing activated more than 60% of %MVIC at bilateral iliopsoas muscles and could be useful as a strength exercise for those muscles. The replaced hip pain related to golf activity was associated with male female, a lower mHHS, and higher HCF of the surgical side; in particular HCF could be reduced through swing adjustments, which may allow for the development of a comfortable golf game free from pain.

## Data Availability

The datasets used and/or analyzed during the current study available from the corresponding author on reasonable request.
